# The Mutational Concordance of Fixed Formalin Paraffin Embedded and Fresh Frozen Gastro-Oesophageal Tumours Using Whole Exome Sequencing

**DOI:** 10.3390/jcm10020215

**Published:** 2021-01-09

**Authors:** Irene Y. Chong, Naureen Starling, Alistair Rust, John Alexander, Lauren Aronson, Marta Llorca-Cardenosa, Ritika Chauhan, Asif Chaudry, Sacheen Kumar, Kerry Fenwick, Ioannis Assiotis, Nik Matthews, Ruwaida Begum, Andrew Wotherspoon, Monica Terlizzo, David Watkins, Ian Chau, Christopher J. Lord, Syed Haider, Sheela Rao, David Cunningham

**Affiliations:** 1 The Division of Molecular Pathology, The Institute of Cancer Research, 237 Fulham Road, London SW3 6JB, UK; lauren.aronson@icr.ac.uk (L.A.); marta.llorca@icr.ac.uk (M.L.-C.); 2 The Royal Marsden Hospital NHS Foundation Trust, 203 Fulham Road, London SW3 6JJ, UK; Naureen.starling@rmh.nhs.uk (N.S.); asif.chaudry@rmh.nhs.uk (A.C.); sacheen.kumar@rmh.nhs.uk (S.K.); ruwaida.begum@rmh.nhs.uk (R.B.); andrew.wotherspoon@rmh.nhs.uk (A.W.); monica.terlizzo@rmh.nhs.uk (M.T.); david.watkins@rmh.nhs.uk (D.W.); ian.chau@rmh.nhs.uk (I.C.); sheela.rao@rmh.nhs.uk (S.R.); david.cunningham@rmh.nhs.uk (D.C.); 3 The Tissue Profiling Unit, The Institute of Cancer Research, 237 Fulham Road, London SW3 6JB, UK; alistair.rust@icr.ac.uk (A.R.); ritika.chauhan@icr.ac.uk (R.C.); kerry.fenwick@icr.ac.uk (K.F.); ioannis.assiotis@icr.ac.uk (I.A.); nik.matthews@icr.ac.uk (N.M.); 4 Breast Cancer Now Toby Robins Research Centre, The Institute of Cancer Research, London SW3 6JB, UK; John.Alexander@icr.ac.uk (J.A.); Chris.lord@icr.ac.uk (C.J.L.); Syed.Haider@icr.ac.uk (S.H.)

**Keywords:** gastro-oesophageal cancer, mutational concordance, exome sequencing, formalin fixed paraffin embedded, biomarkers

## Abstract

1. Background: The application of massively parallel sequencing has led to the identification of aberrant druggable pathways and somatic mutations within therapeutically relevant genes in gastro-oesophageal cancer. Given the widespread use of formalin-fixed paraffin-embedded (FFPE) samples in the study of this disease, it would be beneficial, especially for the purposes of biomarker evaluation, to assess the concordance between comprehensive exome-wide sequencing data from archival FFPE samples originating from a prospective clinical study and those derived from fresh-frozen material. 2. Methods: We analysed whole-exome sequencing data to define the mutational concordance of 16 matched fresh-frozen and FFPE gastro-oesophageal tumours (*N* = 32) from a prospective clinical study. We assessed DNA integrity prior to sequencing and then identified coding mutations in genes that have previously been implicated in other cancers. In addition, we calculated the mutant-allele heterogeneity (MATH) for these samples. 3. Results: Although there was increased degradation of DNA in FFPE samples compared with frozen samples, sequencing data from only two FFPE samples failed to reach an adequate mapping quality threshold. Using a filtering threshold of mutant read counts of at least ten and a minimum of 5% variant allele frequency (VAF) we found that there was a high median mutational concordance of 97% (range 80.1–98.68%) between fresh-frozen and FFPE gastro-oesophageal tumour-derived exomes. However, the majority of FFPE tumours had higher mutant-allele heterogeneity (MATH) scores when compared with corresponding frozen tumours (*p* < 0.001), suggesting that FFPE-based exome sequencing is likely to over-represent tumour heterogeneity in FFPE samples compared to fresh-frozen samples. Furthermore, we identified coding mutations in 120 cancer-related genes, including those associated with chromatin remodelling and Wnt/β-catenin and Receptor Tyrosine Kinase signalling. 4. Conclusions: These data suggest that comprehensive genomic data can be generated from exome sequencing of selected DNA samples extracted from archival FFPE gastro-oesophageal tumour tissues within the context of prospective clinical trials.

## 1. Introduction

Gastric and oesophageal cancers are, respectively, the third and seventh leading causes of cancer-related deaths [1,2,3]. Disease relapse following first-line treatment in patients with advanced disease is frequent, with limited subsequent treatment options. Previously studied targeted therapies in patients with advanced gastro-oesophageal cancer include inhibitors of erythroblastic oncogene B (ERBB2) [4], epidermal growth factor receptor (EGFR) [5,6], vascular endothelial growth factor (VEGF) [7], vascular endothelial growth factor receptor (VEGFR2) [8,9], and poly (ADP-ribose) polymerase (PARP) [10]. However, an improved understanding of individual patient responses is required to identify actionable mechanisms of treatment response and resistance. Genome-wide DNA sequencing studies have confirmed that gastro-oesophageal adenocarcinomas are highly mutated and heterogeneous tumours [11,12]. We and others have identified aberrant druggable pathways and somatic mutations within therapeutically relevant genes in the treatment of naïve frozen gastro-oesophageal tumours using massively parallel sequencing techniques [11,13,14,15]. For the purposes of biomarker evaluation, it would be beneficial to utilise whole-exome DNA sequencing to generate comprehensive genomic data that could be compared with clinical response and outcome within mature phase III studies. Unfortunately, only formalin-fixed paraffin-embedded (FFPE) tissues are available for genomic evaluation in most of these trials; this could potentially be problematic as the process of tissue immobilisation by the FFPE process can result in cross-linked and fragmented DNA that may not be fit for purpose for massively parallel sequencing [16]. It is, therefore, important to understand the level of mutational concordance between frozen and FFPE tumours to assess the utility of next-generation sequencing of DNA extracted from FFPE tissues. Here, we describe an analysis of whole-exome sequencing data to define the mutational concordance of DNA extracted from matched fresh frozen and FFPE gastro-oesophageal tumours, and to estimate the feasibility of this approach within the context of prospective clinical trials.

## 2. Experimental Section

### 2.1. Sample Description and Preparation

Snap frozen and matched FFPE gastro-oesophageal tumour biopsies used for exome sequencing were obtained from patients at the time of endoscopic ultrasound staging, prior to treatment by the same endoscopist at the Royal Marsden Hospital, UK. The biopsies were fixed in neutral buffered formalin for 5–8 h. Oesophageal tumour samples with malignant cell purities of over 70% were selected for DNA extraction and subsequent whole-exome sequencing. Signed written informed consent from each patient was obtained before recruitment to the study according to regulations of the local ethics review board. 

### 2.2. Genomic DNA Extraction and Whole-Exome

Genomic DNA was isolated from tumour biopsies using the DNeasy Blood and Tissue kit (Qiagen, Hilden, Germany) and quantified using Qubit fluorometric quantitation (Invitrogen Life Technologies, Carlsbad, CA, USA). Genomic DNA was fragmented to 200 basepairs (bps) using a Covaris E Series instrument (Covaris Inc., Woburn, MA, USA). The resultant library was subjected to DNA capture using the 50 Mb SureSelect Human All Exon V5 kit (Agilent, Santa Clara, CA, USA). DNA capture was carried out, and Illumina paired-end libraries were prepared from the captured target regions and quantified using a Bioanalyzer DNA chip (Agilent). This process was then followed by sequencing on a HiSeq2500 platform (Illumina, San Diego, CA, USA), acquiring 2 × 100 bps reads. Bcl2fastq software (v1.8.4, Illumina) was used for converting the raw basecalls to fastqs and to further demultiplex the sequencing data. The demultiplexed paired-end fastq files were used for further analysis.

### 2.3. Read Mapping and Detection of Mutations from Exome Sequencing 

BWA-mem (v0.7.5a) was used to align reads to the human reference genome (GRCh37) [17]. Variant calling was carried out using the Broad Best Practice pipeline with standard settings [18]. In summary, GATK (v3.3-0) was used to detect frameshifts and MuTect (v1.1.4) was used to detect point mutations. The effects of single-point mutations were determined by SnpEff (v3.3h). Candidate mutations were selected using the following list of heuristic rules: (1) variants detected at a mutant allele frequency (MAF) of greater than 5% in any of the 1000 Genomes project populations were excluded from analysis, (2) variants called in regions not covered by the exome capture probes were excluded, (3) variants marked as low quality (QUAL below 30) were excluded, and (4) variants not reaching a depth threshold of 10 reads were excluded.

## 3. Results

### 3.1. Clinicopathological Features of Patients

All patients were treatment-naïve at the time of biopsy retrieval. The median age was 64 years for the 16 patients included in this study (Table 1). The majority were male (81.2%). The most common disease site was at the gastro-oesophageal junction (GOJ, 68.8%). The GOJ and gastric tumours were adenocarcinomas (93.8%) that were either moderately or poorly differentiated (grade 2 or 3). The remaining cancer was an early, well-differentiated (grade 1) neuroendocrine tumour located in the distal oeosphagus. The majority of tumours were locally advanced (T3 N0/1 M0, 62%). Four patients had early disease (T1/2 N0, M0, 25%), and two patients presented with metastatic disease (T3 N1 M1, 12.5%). The storage period of the tissues ranged from 4 to 10 years, with a median time of 8.5 years. 

### 3.2. Assessment of DNA Integrity

**Figure 1:**
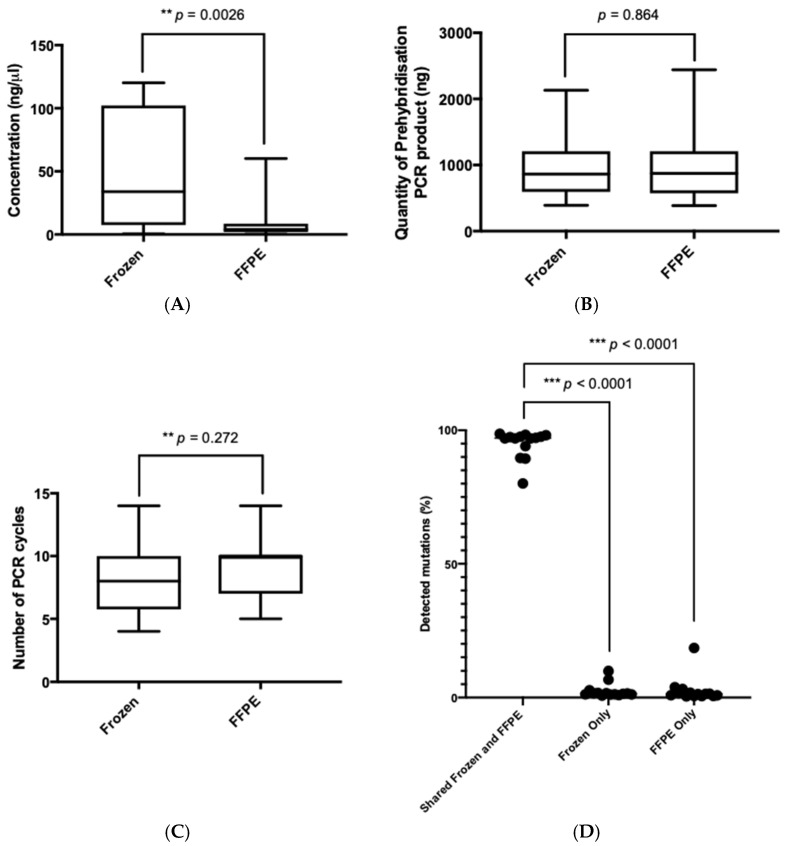
Mutational concordance between frozen and formalin-fixed paraffin-embedded (FFPE) gastro-oesophageal tumour samples. Box and whiskers plots showing the distribution of (**A**). The concentration of double-stranded DNA (nanograms per microlitre) extracted from FFPE and frozen gastro-oesophageal tumours. An increased double-stranded DNA yield was extracted from frozen tumour tissues compared with FFPE tissue (*p* = 0.0026,) (**B**). Prehybridisation PCR product (nanograms) (**C**). The number of PCR cycles required to generate the pre-hybridisation library from FFPE and frozen gastro-oesophageal tumour samples. No difference between FFPE and frozen samples was observed in terms of the overall quantity of pre-hybridisation PCR product generated, nor in terms of the number of PCR cycles required to generate the pre-hybridisation library prior to sequencing (**D**). Bar graph showing a high mutational concordance (range 80.1% to 98.68%) in terms of the percentage of shared mutations detected (in both frozen and FFPE samples) compared with mutations unique to frozen samples and FFPE samples.

We observed that there was a significant difference in the concentration of double-stranded DNA extracted from frozen compared with FFPE oesophageal tumour (*p* = 0.0026, Mann-Whitney U test), suggesting improved integrity of DNA extracted from frozen samples and increased degradation of FFPE biospecimens (Figure 1A) [19]. However, there was no significant difference in either the total quantity of pre-hybridisation PCR product generated or the number of PCR cycles required to generate the pre-hybridisation library prior to exome sequencing (Figure 1B,C). Following exome sequencing, mutation filtering was applied including mapping quality threshold of ≥30, depth threshold of ≥10 reads, and variant allele frequency (VAF) threshold of ≥0.05. Of note, in the absence of matched blood samples, many germline variants are likely to exist in our mutational repertoire. By applying these thresholds, mutation calls detected in frozen tumour samples were considered a gold standard, allowing for the calculation of true positive, false positive, and false negative rates. For each set of thresholds, combined numbers for sensitivity, precision/positive predictive value (PPV), and F-score were calculated (Table 2). The two sets of thresholds with the highest PPV and F-scores were for mutant read counts of ten or more and a minimum of 5% VAF. We observed that all of the 16 frozen samples achieved adequate exome coverage and depth. However, two of the 16 FFPE samples (samples 178 and 260) did not achieve the minimum median depth threshold of 50×. The ages of the two FFPE specimens that failed were 5 years and 10 years, respectively (the range for this cohort was 4–10 years). Whilst the initial starting quantities of DNA and following fragmentation were adequate, the total amount of post-adapter-ligation DNA was lower than expected (less than 400 ng), indicating inferior DNA quality. These samples failed the quality control criteria and were excluded from further analyses. 

### 3.3. Mutational Concordance between Frozen and FFPE Oesophageal Tumour Samples

To assess the mutational concordance between matched frozen and FFPE-derived gastro-oesophageal tumour DNA in the 14 matched samples that passed quality control criteria (*N* = 28), we cross-referenced mutations detected from exome sequencing. We observed that there was a high median mutational concordance of 97.07% (range 80.1% to 98.68%) between fresh-frozen and FFPE gastro-oesophageal tumour samples (Figure 1D, Table 3). There was no difference overall in the percentage of unique mutations found in DNA derived from FFPE compared with frozen tumour tissue (*p* = 0.41, Mann–Whitney U test). Given that 93% (90/96) of randomly selected mutations have previously been validated with Sanger sequencing, and that 95% (1791/1883) of mutations were recognised by both exome sequencing and the Ion Proton platform from our previous study [14], our current results demonstrate the feasibility of exome sequencing of FFPE-derived DNA samples from gastro-oesophageal tumours that have passed the described quality control criteria.

### 3.4. Detection of Mutations within Cancer-Related Genes

**Figure 2:**
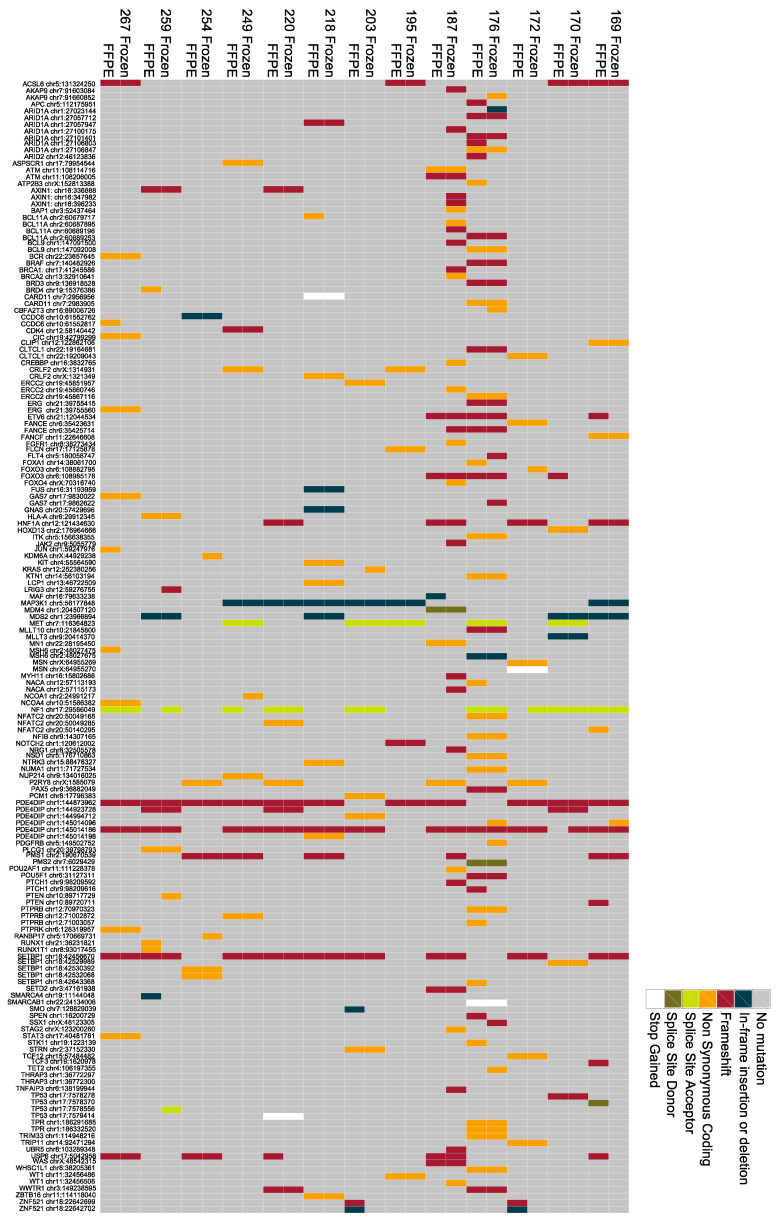
Detection of cancer-related genes from exome sequencing of gastro-oesophageal tumour DNA. Commutation plot showing the presence of mutations in genes from the Cancer Genome Census from exome sequencing of DNA extracted from frozen and formalin-fixed paraffin-embedded (FFPE) oesophageal tumours.

To identify coding mutations in genes from exome sequencing that have also been implicated in other cancers, we correlated genes harbouring frameshift, non-synonymous, splice site, and stop-gained mutations with genes in the Cancer Genome Census (CGC) [20]. Overall, this comparison identified 120 cancer-related genes in the gastro-oesophageal samples from this study, with an average of 12 potentially deleterious CGC mutations (range 7–50) present in each sample (Figure 2). These mutations were further analysed to determine the dysregulation of cancer-associated pathways. Using this approach, we observed coding mutations in tumour-suppressor genes usually required for normal chromatin remodelling, including *ARID1A* (AT-rich interaction domain 1A gene), *BRD3* (Bromodomain-containing protein 3 gene), and *SMARCA4* (SWI/SNF-Related Matrix-Associated Actin-Dependent Regulator of Chromatin Subfamily A, Member 4 gene). In addition, 8 out of 16 tumours harboured mutations in well-established DNA repair-related tumour-suppressor genes, including *FANCE* (Fanconi Anemia Complementation Group E gene), *FANCF* (Fanconi Anaemia Complementation Group F gene), *MSH6* (MutS Homolog 6 gene), *PMS1* (PMS1 Homolog1, Mismatch Repair System Component gene), *PMS2* (PMS1 Homolog 2, Mismatch Repair System Component gene), *ERCC2* (ERCC Excision Repair 2, TFIIH Core Complex Helicase Subunit gene), or *SETD2* (SET Domain Containing 2 gene), suggestive of disrupted DNA repair pathway signalling in these tumours. Coding mutations in genes involved in Wnt signalling were detected, including mutations in *BCL9* (B-Cell CLL/Lymphoma 9 gene) and *AXIN1* (Axin 1 gene). Coding mutations in *TP53* were detected in 4 out of 16 tumours from exome sequencing. We also identified mutations in genes involved in RAS/RAF signalling, including *KRAS* (Kirsten ras oncogene) and *BRAF* (B-Raf Proto-Oncogene, Serine/Threonine Kinase gene). Mutations in therapeutically relevant genes were also observed, including those in *MET* (MET Proto-Oncogene, Receptor Tyrosine Kinase gene) and *FGFR1* (Fibroblast Growth Factor Receptor 1 gene). 

### 3.5. Intratumoural Genetic Heterogeneity

**Figure 3:**
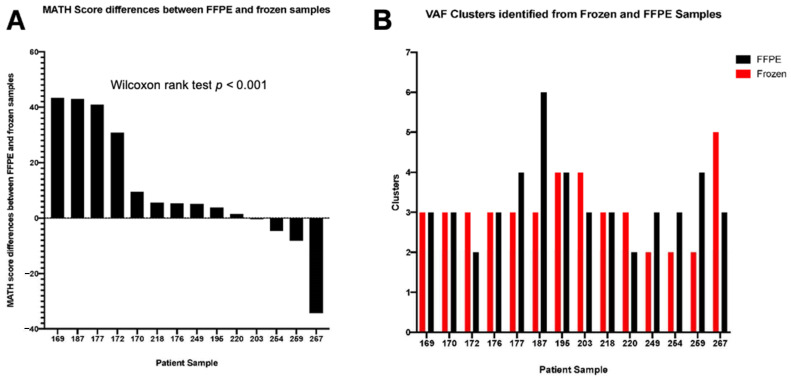
Differences in mutant-allele heterogeneity (MATH) scores and variant allele frequency (VAF) clusters identified from matched formalin-fixed paraffin-embedded (FFPE) and frozen gastro-oesophageal tumours. Histogram illustrating (**A**) differences in MATH scores between FFPE and frozen tumour samples—11 out of 14 FFPE tumours had higher MATH scores when compared with the corresponding frozen tumours (*p* < 0.001 Wilcoxon rank test); and (**B**) VAF clusters identified from FFPE and frozen samples.

Gastro-oesophageal tumours are known to be heterogeneous cancers [14,21]. Intratumoural heterogeneity with respect to actionable mutations has clinical implications for how targeted therapies might work [22,23]. Genomically distinct subpopulations of cells lead to differences among mutated loci in terms of the fraction of sequence reads displaying a mutant allele. A heterogeneous tumour will likely have a wider distribution of mutant-allele fractions among loci centred at a lower fraction, compared with a homogeneous tumour [24]. Taking this into consideration, we analysed exome sequencing results for each of the frozen and FFPE tumours. Moreover, we calculated the mutant-allele heterogeneity (MATH) score as the ratio of the width to the centre of its distribution of mutant-allele fractions among tumour-specific mutated loci (Supplementary Figure S1). We observed that the median MATH score for the frozen tumours was 32.95 (range 17.4 to 96.6), indicating notable differences in inter-tumoural heterogeneity in this set of gastro-oesophageal samples. The majority of the FFPE tumours (11 out of 14 samples) had higher MATH scores when compared with the corresponding frozen tumours (*p* < 0.001 Wilcoxon rank test, Figure 3A), suggesting that this analysis is likely to over-represent tumour heterogeneity in FFPE samples. In addition, the number of clonal clusters calculated by MATH was discordant in 9 out of 14 matched samples (Figure 3B). Although the median mutational concordance between fresh-frozen and FFPE gastro-oesophageal tumour samples was high (median 97%, range 80.1–98.68%), MATH analysis to assess tumour heterogeneity was not found to be reliable in FFPE samples.

## 4. Discussion

Considering the potential clinical impact of dissecting molecular mechanisms of treatment response and resistance within prospective clinical trials where only FFPE samples are available for analysis [25], the main purpose of this study was to assess the feasibility of using DNA extracted from FFPE gastro-oesophageal tumour for massively parallel sequencing. It is acknowledged that DNA cross-linking, degradation, and fragmentation occurring during the FFPE process has the potential to influence the reliability of mutational sequencing data [26,27,28,29]. Taking matched FFPE and frozen melanoma specimens as examples, a comparison of whole-exome sequencing data from 10 tumours revealed a very low overall mutational concordance (average 43.2%). However, the most clinically actionable mutations for this tumour type (*BRAF* and *NRAS*) were found to be concordant [30]. The authors from this study concluded that specialised library construction to account for low quality DNA is necessary before this approach could be used for routine clinical decision making. In contrast, studies relating to other tumour types and utilizing different massively parallel sequencing techniques have yielded more promising results; the concordance rate was found to be up to 96.8% in a lung cancer study comparing the variants of 27 cancer-related genes in 16 matched FFPE and frozen samples [31]. Mutational comparisons have also been undertaken in colorectal cancer (CRC) specimens; the detected concordance rate was up to 81.9% in a study of 33 matched metastatic CRC samples [32]. In a cohort of 10 paired metastatic liver CRC specimens, a high mutational concordance was observed when 212 amplicon regions in 48 cancer-related genes were sequenced, revealing 21 identical mutation calls and only two differing mutations [33]. Furthermore, Gao et al. conducted an extensive study using a 22-gene panel detecting 103 hotspot mutations in paired FFPE and fresh-frozen primary CRC tissues from 118 patients [34]. The investigators identified a concordance rate ranging from 73.8% to 100% and highlighted that important differences exist between the two tissue types. 

We approached this problem by assessing DNA integrity prior to sequencing and analysing whole-exome sequencing data to define the mutational concordance of matched fresh-frozen and FFPE gastro-oesophageal tumours. As expected, DNA degradation was more pronounced in the FFPE biospecimens compared with the matched frozen samples. However, there was no significant differences in either the total quantity of pre-hybridisation PCR product generated or the number of PCR cycles required to generate the pre-hybridisation library prior to exome sequencing. Only two out of 16 FFPE samples failed quality control criteria with the inability to achieve the minimum median depth threshold of 50×. In the absence of normal/germline samples, we considered all variants likely to include many germline variants. Based on these variants, the subsequent calculation of PPV and F-scores, allowing for the calculation of true-positive, false-positive, and false-negative rates, using frozen tumour samples as a gold standard, identified the optimal filtering threshold as mutant read counts of 10 or more and a minimum of 5% VAF. Using this threshold, we observed a high median mutational concordance of 97% between DNA derived from fresh frozen and FFPE gastro-oesophageal tumours. Consistent with the literature, we also identified frequent mutations in genes responsible for chromatin remodelling, Wnt/β-catenin, and Receptor Tyrosine Kinase signalling [13]. Finally, we assessed intratumoural heterogeneity by calculating the MATH score, and the ratio of the width to the centre of its distribution of mutant-allele fractions among tumour-specific mutated loci, for each sample. We found that most FFPE gastro-oesophageal tumours in this study had higher MATH scores compared with the corresponding frozen tumours. FFPE samples are likely to over-estimate tumour heterogeneity due to the presence of artefactual substitutions in FFPE samples [35]. This result may lead to a more significant variation in observed VAFs, resulting in a higher MATH score. 

Focusing on the two FFPE specimens that failed sequencing quality control, we have scrutinised the clinicopathological characteristics of patients included in this study, as well as the raw data generated after DNA extraction and before massively parallel sequencing, to evaluate whether, at any stage, sequencing failure could have been predicted. We found that none of the clinical characteristics were responsible. In particular, the age of the two FFPE specimens that failed were five years and ten years, respectively (the range for this cohort was 4–10 years). Furthermore, we confirmed that the initial quantities of DNA and following fragmentation were indeed adequate. However, the total amount of post-adapter-ligation DNA was lower than expected (less than 400 ng), which is an indication of inferior DNA quality. Whilst this finding could serve as a warning for investigators, we cannot definitively conclude that this factor alone should preclude the commencement of exome sequencing in future studies. 

Our findings support the validity of massively parallel sequencing of FFPE gastro-oesophageal tissues as a discovery tool, recognising that only archival tumour blocks are available in the majority of completed phase III studies. Through rigorous assessment of DNA integrity and application of an optimal filtering threshold, a high level of mutational concordance between FFPE and frozen tissues can be achieved. However, subsequent orthogonal validation of actionable mutations is of utmost importance. In contrast, the assessment of intratumoural heterogeneity using the distribution of mutant allele fractions in FFPE gastro-oesophageal samples is much less reliable.

## Figures and Tables

**Table 1:** Clinicopathological characteristics of patients.

Characteristic	(*N* = 16)
Age at diagnosis	
Median—y	64
Range—y	22–82
Sex—No. (%)	
Male	13 (81.2)
Female	3 (18.8)
Site of tumour—No. (%)	
Distal oesophagus	1 (6.3)
GOJ type I	3 (18.7)
GOJ type II	4 (25)
GOJ type III	4 (25)
Stomach	4 (25)
Histology—No. (%)	
Adenocarcinoma	15 (93.8)
Neuroendocrine	1 (6.2)
Grade—No. (%)	
1	1 (6.2)
2	5 (31.3)
3	10 (62.5)
TNM Stage—No. (%)	
T1/2 N0 M0	4 (25)
T3 N0/1 M0	10 (62.5)
T3 N1 M1	2 (12.5)
Time from biopsy to sequencing	
Median—y	8.5
Range—y	4–10

**Table 2:** Sensitivity, precision/positive predictive value (PPV), and F-Score for selected variant allele frequency (VAF) and tumour depth thresholds.

VAF (%)	Tumour Depth (X)	Combined Sensitivity	Combined Precision PPV	Combined F Score
2	5	0.775700935	0.83	0.801932367
5	5	0.775700935	0.83	0.801932367
10	5	0.76076555	0.81122449	0.785185185
15	5	0.712643678	0.765432099	0.738095238
20	5	0.732283465	0.801724138	0.765432099
2	10	0.778301887	0.829145729	0.802919708
5	10	0.778301887	0.829145729	0.802919708
10	10	0.763285024	0.81025641	0.786069652
15	10	0.715116279	0.763975155	0.738738739
20	10	0.744	0.801724138	0.771784232
2	15	0.773584906	0.83248731	0.80195599
5	15	0.773584906	0.83248731	0.80195599
10	15	0.758454106	0.813471503	0.785
15	15	0.709302326	0.767295597	0.737160121
20	15	0.736	0.807017544	0.769874477
2	20	0.763033175	0.829896907	0.795061728
5	20	0.763033175	0.829896907	0.795061728
10	20	0.747572815533981	0.810526316	0.777777778
15	20	0.695906433	0.762820513	0.727828746
20	20	0.717741935	0.801801802	0.757446809
2	25	0.759615385	0.822916667	0.79
5	25	0.759615385	0.822916667	0.79
10	25	0.748768473	0.808510638	0.777493606
15	25	0.704142012	0.767741935	0.734567901
20	25	0.729508197	0.809090909	0.767241379
2	30	0.747572816	0.814814815	0.779746835
5	30	0.747572816	0.814814815	0.779746835
10	30	0.736318408	0.8	0.766839378
15	30	0.694610778	0.753246753	0.722741433
20	30	0.716666667	0.788990826	0.751091703

**Table 3:** The of percentage mutational concordance of matched fresh frozen and formalin-fixed paraffin-embedded (FFPE) gastro-oesophageal tumour samples.

Patient	Sample	Mutations Unique to Sample	% Unique to Sample
169	Frozen	1955	6.72
	FFPE	1129	3.88
	Shared	25,997	89.40
170	Frozen	456	1.65
	FFPE	250	0.90
	Shared	26,958	97.45
172	Frozen	471	1.73
	FFPE	361	1.32
	Shared	26,424	96.95
176	Frozen	853	2.72
	FFPE	1017	3.24
	Shared	29,508	94.04
177	Frozen	476	1.42
	FFPE	6205	18.48
	Shared	26,896	80.10
187	Frozen	3056	9.91
	FFPE	146	0.47
	Shared	27,630	89.61
195	Frozen	204	0.72
	FFPE	168	0.60
	Shared	27,790	98.68
203	Frozen	428	1.56
	FFPE	233	0.85
	Shared	26,811	97.59
218	Frozen	311	1.14
	FFPE	154	0.57
	Shared	26,738	98.29
220	Frozen	317	1.18
	FFPE	493	1.84
	Shared	25,978	96.98
249	Frozen	257	0.92
	FFPE	408	1.47
	Shared	27,119	97.61
254	Frozen	327	1.23
	FFPE	159	0.60
	Shared	26,076	98.17
259	Frozen	325	1.20
	FFPE	441	1.63
	Shared	26,234	97.16
267	Frozen	468	1.61
	FFPE	413	1.42
	Shared	28,209	96.97

## Data Availability

The raw data supporting the conclusions of this article are available on request to the corresponding author.

## Supplementary Materials

The following are available online at https://www.mdpi.com/2077-0383/10/2/215/s1, Figure S1: MATH Profiles of intra-tumoural heterogeneity in matched grozen and FFPE gastro-oesophageal tumours.
