# Supplementary material for: The Mutational Concordance of Fixed Formalin Paraffin Embedded and Fresh Frozen Gastro-Oesophageal Tumours Using Whole Exome Sequencing
Source: J Clin Med. 2021 Jan 9;10(2):215. doi: 10.3390/jcm10020215 (PMC7826535; doi:10.3390/jcm10020215)
Supplement: Supplementary file 1 [file jcm-10-00215-s001.pdf]

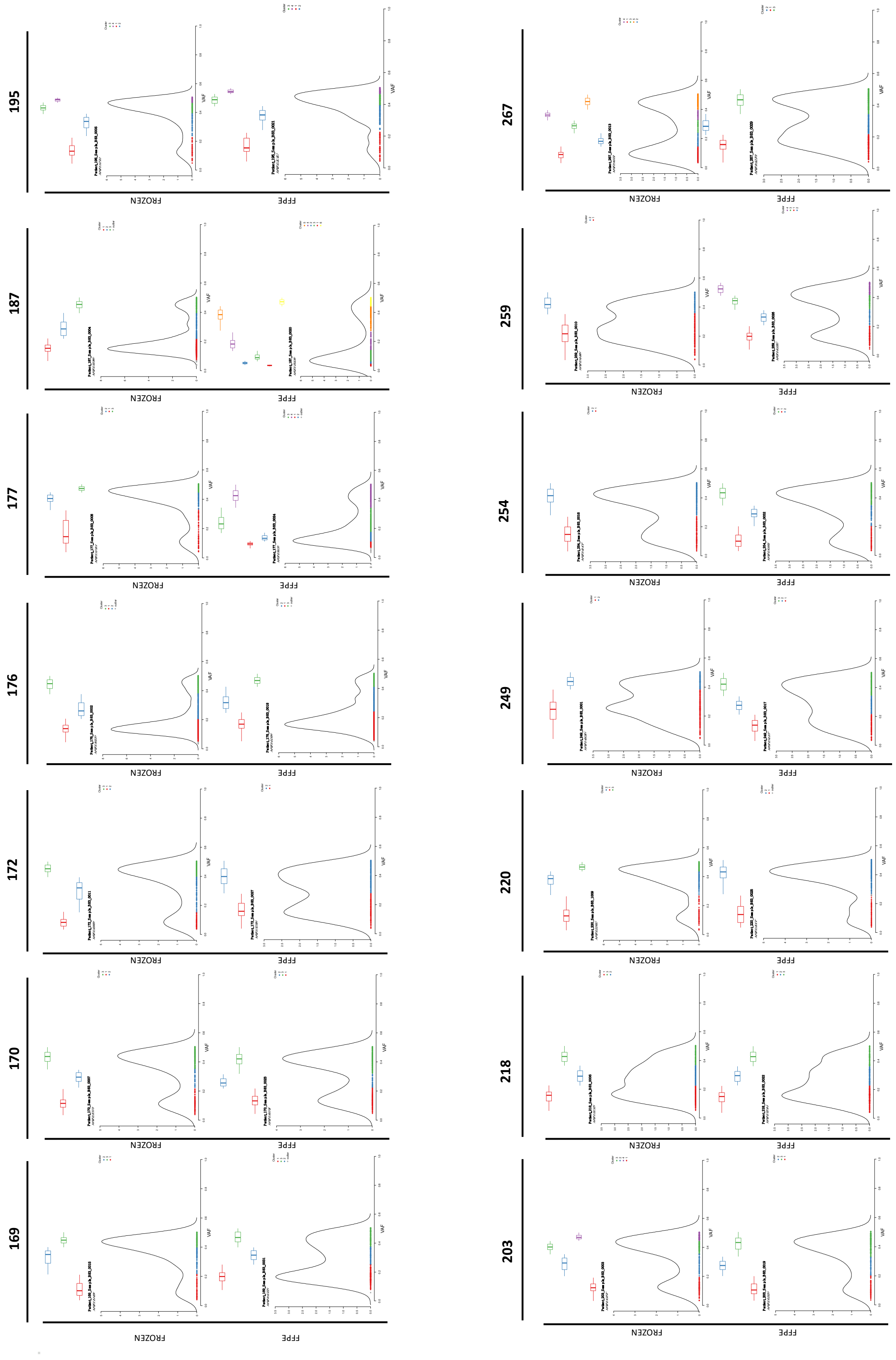

Supplementary Figure 1. MATH Profiles of Intra-tumoural Heterogeneity in Matched Frozen and FFPE Gastro-oesophageal Tumours.
